# Case Report: Computed tomographic diagnosis of asymmetric bilateral uterine horn torsion secondary to benign uterine leiomyomas in a non-gravid dog

**DOI:** 10.3389/fvets.2026.1959325

**Published:** 2026-09-25

**Authors:** Eunseok Jeong, Jaewon Kim, Younghwan Kim, Kidong Eom, Jaehwan Kim

**Affiliations:** 1 Department of Veterinary Medical Imaging, College of Veterinary Medicine, Konkuk University, Seoul, Republic of Korea; 2 Royal Animal Medical Center, Seoul, Republic of Korea

**Keywords:** case report, computed tomography, leiomyoma, uterine torsion, whirl sign

## Abstract

Uterine torsion is uncommon in dogs and most commonly involves a single uterine horn. An 11-year-old intact female Pomeranian was presented with a 2- to 3-day history of abdominal distension, dyspnea, anorexia, vomiting, and oliguria. Clinicopathologic findings indicated renal compromise, electrolyte abnormalities, tissue injury, and marked systemic inflammation. Radiography and ultrasonography revealed large bilateral abdominal masses with peritoneal effusion, although the organ of origin could not be determined. Contrast-enhanced computed tomography (CT) demonstrated masses arising from both uterine horns, with absent central enhancement on the right, minimal central enhancement on the left, and thin peripheral rim enhancement of both masses, suggesting vascular compromise and ischemic injury. Both ovaries showed preserved contrast enhancement, and spiral twisting of both uterine horns and bilateral whirl signs were identified, with the changes being markedly more pronounced on the right. These findings permitted a preoperative diagnosis of asymmetric bilateral uterine horn torsion. Exploratory laparotomy confirmed approximately 1,080° rotation of the right horn around the left horn and 270° rotation of the left horn around its own axis, without gross rupture of the broad ligament. En bloc ovariohysterectomy without prior detorsion and excision of the vaginal mass were performed. Histopathologic examination confirmed leiomyomas of both uterine horns and of the vaginal mass, with extensive hemorrhage and necrosis of the right horn. The dog recovered uneventfully and remained clinically well 9 months after surgery. To the authors’ knowledge, this is the first report of bilateral uterine torsion associated with benign leiomyomas in a dog and the first description of bilateral whirl signs on CT in canine uterine torsion.

## 1 Introduction

Uterine torsion is an uncommon but potentially life-threatening condition in dogs. It is most often reported in the periparturient period, particularly during late gestation (1). In non-gravid animals, the principal predisposing factors are conditions that increase uterine size and weight, including hematometra, pyometra, cystic endometrial hyperplasia, endometrial polyps, and uterine neoplasia such as leiomyosarcoma and lipoma (2–5). In most reported cases, torsion involves a single uterine horn (4, 6). Bilateral uterine torsion is exceedingly rare and has been described in only one previous canine report, in which the two horns were intertwined (7).

Leiomyoma is the most common uterine tumor in dogs (8), and uterine leiomyomas of considerable size have been reported in this species (9). Tumor-associated uterine torsion has previously been reported in dogs only with leiomyosarcoma and lipoma (2, 5), and not with benign leiomyoma.

Preoperative diagnosis remains difficult because radiographic and ultrasonographic findings are non-specific, and definitive diagnosis has traditionally required surgical exploration. In human medicine, computed tomography (CT) may facilitate the preoperative diagnosis of uterine torsion by demonstrating twisting of the vascular pedicle, reduced internal enhancement, peripheral rim enhancement, and hypoenhancement of the adjacent uterine tissue (10, 11). To the authors’ knowledge, bilateral uterine torsion secondary to benign leiomyomas has not previously been reported in dogs, nor have bilateral whirl signs been described on CT in canine uterine torsion.

This report describes such a case and highlights the value of CT in guiding timely surgical management.

## 2 Case description

An 11-year-old intact female Pomeranian weighing 5.42 kg, with a body condition score of 5/9, was presented with a 2- to 3-day history of abdominal distension, dyspnea, anorexia, vomiting, and oliguria. The dog was nulliparous and had no previous history of reproductive disease and no relevant previous medical or surgical history.

On physical examination, marked abdominal enlargement was noted, with a large palpable intra-abdominal mass and a concurrent vaginal mass. The dog was lethargic and tachypneic, and systolic arterial blood pressure was 105 mmHg after fluid resuscitation.

Serum biochemical analysis revealed azotemia, with increased blood urea nitrogen (51.7 mg/dL; reference range, 5 to 30) and creatinine (2.3 mg/dL; 0.5 to 1.5), together with increased symmetric dimethylarginine (30 μg/dL; 0 to 14), hyperphosphatemia (9.5 mg/dL; 2.4 to 6.4), hyponatremia (128 mmol/L; 139 to 151), and hyperkalemia (6.5 mmol/L; 3.6 to 5.3). Creatine kinase activity was increased (1,079 U/L; 25 to 167), C-reactive protein concentration was markedly increased (132 mg/L; <10), and packed cell volume was mildly decreased (32.4%; 37 to 55). These findings indicated renal compromise, electrolyte derangement, tissue injury, and marked systemic inflammation.

Thoracic and abdominal radiographs were obtained (HF 525Plus VET; Eco Ray, Seoul, Republic of Korea). Abdominal radiographs revealed bilateral soft tissue opaque masses occupying the mid to caudal abdomen, with marked mass effect and diffusely decreased serosal detail, whereas thoracic radiographs were unremarkable (Figures 1A,B). Abdominal ultrasonography (Aplio i700 with an i18LX5 linear transducer at 12 MHz; Canon Medical Systems, Tustin, CA, USA) revealed large bilateral heterogeneous masses with a small volume of peritoneal effusion (Figures 1C,D). Ovarian or uterine neoplasia was favored, but the size of the masses precluded identification of the organ of origin. At this stage, differential diagnoses included ovarian or uterine neoplasia and other uterine proliferative lesions, such as endometrial polyps. Fine-needle aspiration of the abdominal mass and of the vaginal mass yielded clusters of mature spindle cells consistent with a mesenchymal neoplasm, without overt cytologic features of malignancy. Ultrasound-guided abdominocentesis was performed, and the peritoneal effusion was characterized as a modified transudate with neutrophilic inflammation and no cytologic evidence of sepsis.

**Figure 1:**
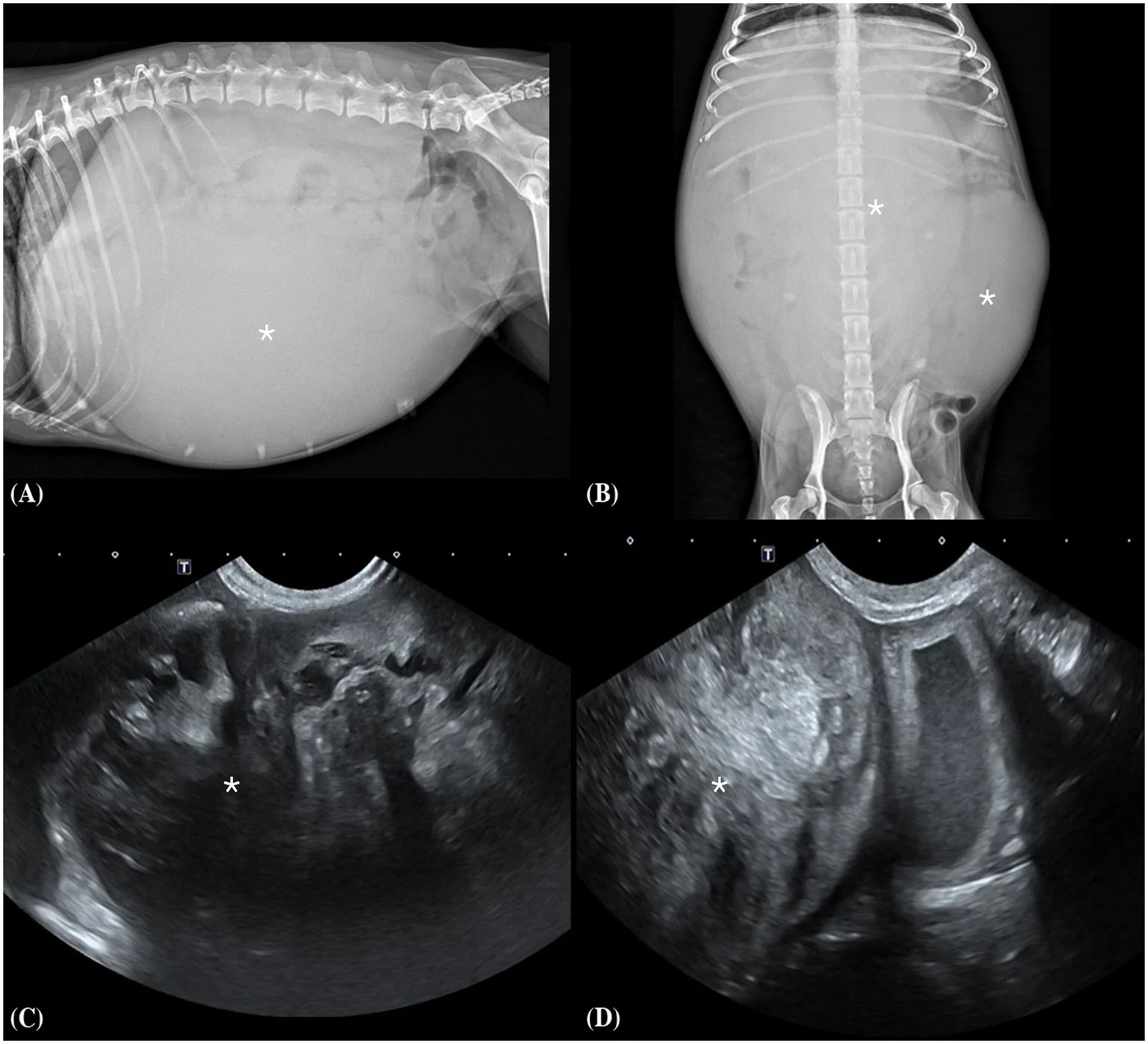
Radiographic and ultrasonographic findings. **(A)** Right lateral and **(B)** ventrodorsal abdominal radiographs showing marked abdominal distension and bilateral soft tissue opaque masses (asterisks). **(C,D)** Ultrasonographic images showing the masses (asterisks) with mildly echogenic peritoneal effusion. Asterisks mark the masses in all panels.

Contrast-enhanced CT (Optima CT660; GE Healthcare, Chicago, IL, USA) was performed under general anesthesia in sternal recumbency, with a tube voltage of 120 kVp, a tube current of 170 mA, a slice thickness of 1.25 mm, and a rotation time of 0.8 s. Unenhanced, arterial (15 s), portal venous (40 s), and delayed (90 s) phase series were acquired after intravenous administration of iohexol (Omnipaque; GE Healthcare, Chicago, IL, USA) at 2.5 mL/kg.

Large soft tissue attenuating masses arose from both uterine horns, with the right substantially larger than the left. The right measured 16.6 × 8.9 × 11.3 cm and the left 11.4 × 7.8 × 5.5 cm. The uterine body and cervix were displaced but remained identifiable, confirming a uterine horn origin. A focal hyperattenuating area within the right mass measured 54 Hounsfield units (HU) on unenhanced images, suggestive of intralesional hemorrhage (Figure 2A). Both masses showed thin peripheral rim enhancement, whereas central enhancement was absent within the right mass and minimal within the left mass (37–40 HU), suggesting vascular compromise and ischemic injury (Figure 2B). Both ovaries were identified, appeared normal in size and morphology, and showed preserved contrast enhancement, indicating that the masses were not of ovarian origin (Figure 2C).

**Figure 2:**
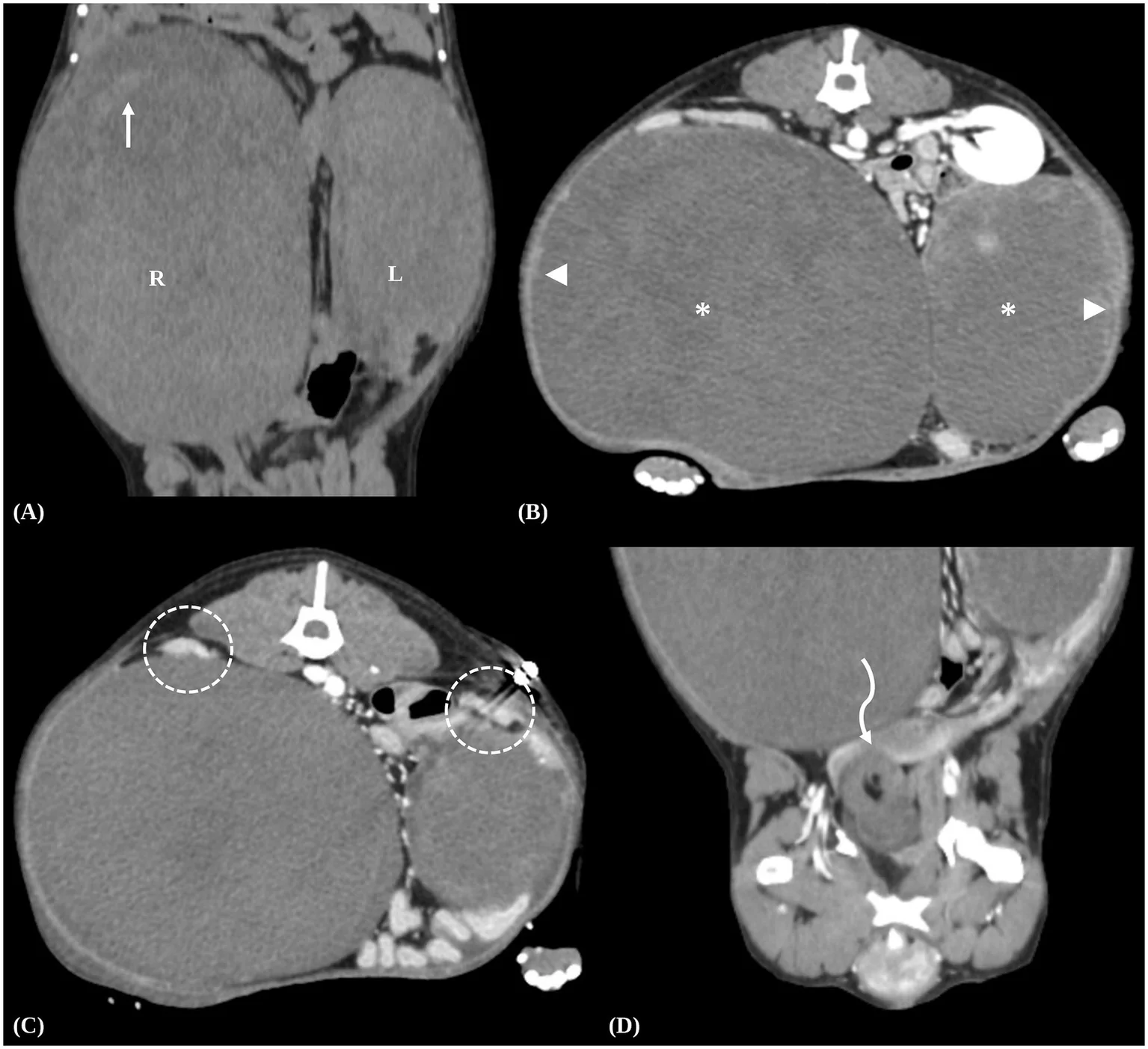
Contrast-enhanced computed tomographic images. **(A)** Unenhanced dorsal image showing the right (R) and left (L) uterine masses, with a focal hyperattenuating area (arrow; 54 HU) within the right mass. **(B)** Delayed-phase transverse image showing thin peripheral rim enhancement (arrowheads) of both masses (asterisks), with absent central enhancement on the right and minimal central enhancement on the left, suggesting different degrees of vascular compromise and ischemic injury. **(C)** Delayed-phase transverse image showing both ovaries (dashed circles) with normal size, morphology, and contrast enhancement, indicating that the masses were not of ovarian origin. **(D)** Delayed-phase dorsal image showing spiral twisting of the distal portion of the right uterine horn (curved arrow). HU, Hounsfield units; R, right; L, left.

Spiral twisting of the distal portions of both uterine horns was evident, with the right horn showing more extensive rotation and twisting around the left horn (Figure 2D). Bilateral whirl signs, representing twisting of the uterine horns, their vascular pedicles, and the surrounding fat around the torsion axes, were identified and were markedly more pronounced on the right (Figures 3A–C). An enhancing mass measuring 2.4 × 2.0 × 2.1 cm was present in the distal vagina. Additional findings included caudodorsal displacement of the urinary bladder, displacement of both kidneys without ureteral dilation, peritoneal effusion measuring 16 HU, and fat stranding around both uterine masses. No lymphadenopathy or evidence of metastatic disease was detected on whole-body CT. These findings enabled a preoperative diagnosis of asymmetric bilateral uterine horn torsion.

**Figure 3:**
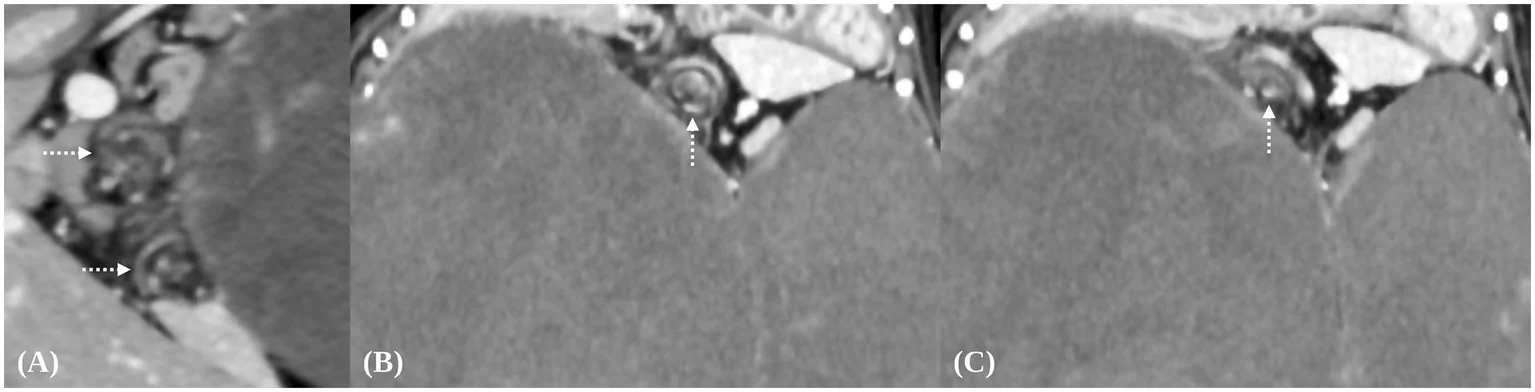
Bilateral whirl signs on computed tomography. **(A)** Sagittal image showing separate whirl signs of the right and left uterine horns (dashed arrows). **(B)** Dorsal image showing the more pronounced whirl sign of the right uterine horn (dashed arrow). **(C)** Dorsal image showing the less pronounced whirl sign of the left uterine horn (dashed arrow), consistent with a greater degree of torsion on the right.

Given the preoperative diagnosis of uterine torsion with suspected severe vascular compromise, exploratory laparotomy was performed without prior detorsion. At surgery, the right uterine horn was rotated approximately 1,080° counterclockwise around the left horn, corresponding to three complete turns, and was markedly congested with a grossly necrotic appearance, whereas the left horn was rotated approximately 270° counterclockwise around its own axis and showed milder changes. The degree of torsion was estimated intraoperatively based on the number and extent of rotations observed during surgical inspection. The right vascular pedicle and broad ligament were also markedly twisted, without gross rupture of the ligament. En bloc ovariohysterectomy and excision of the vaginal mass were performed (Figures 4A,B). On gross sectioning, the right uterine horn was diffusely dark red, suggestive of extensive hemorrhage and necrosis (Figure 4C).

**Figure 4:**
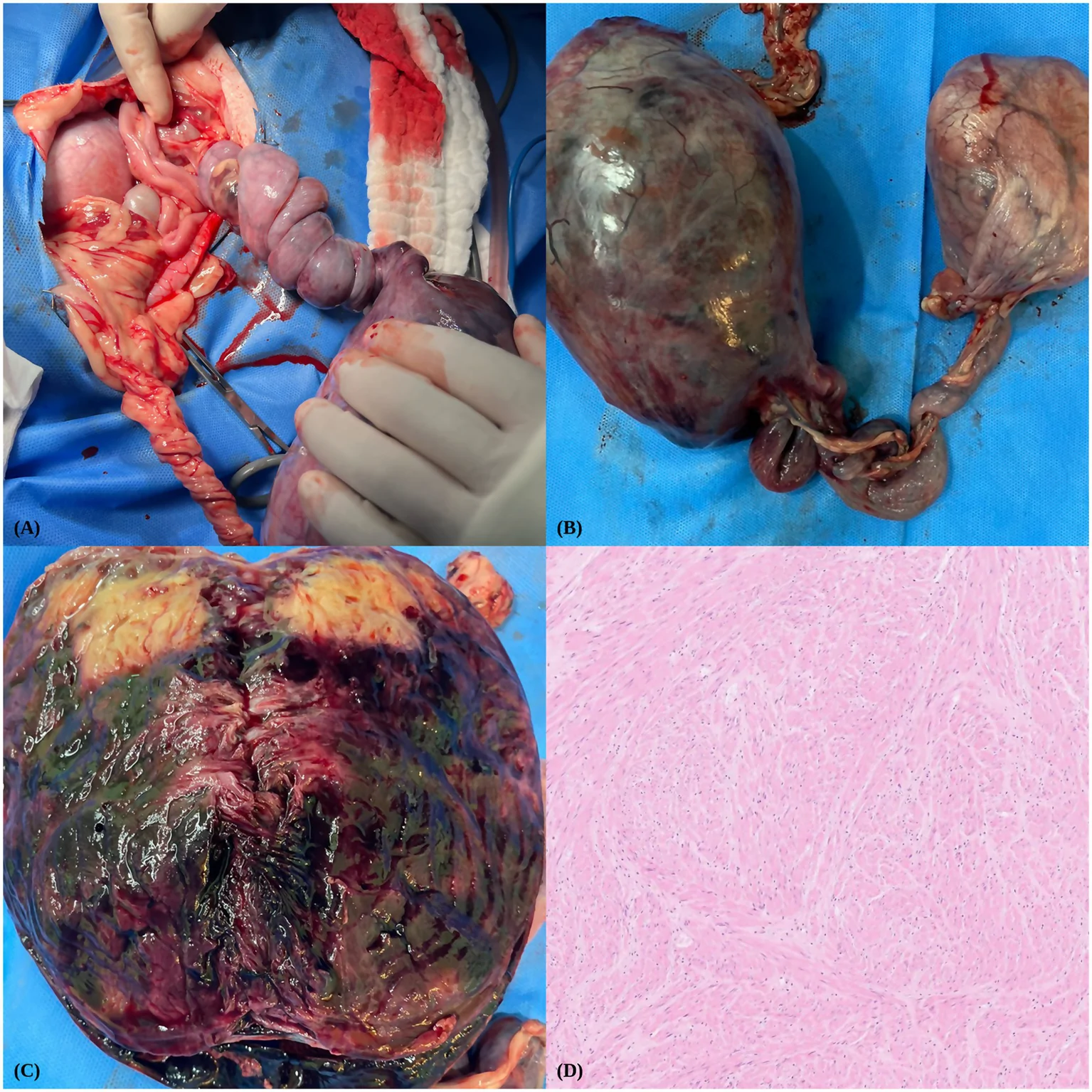
Surgical, gross, and histopathologic findings. **(A)** Intraoperative photograph showing marked twisting of the right vascular pedicle and broad ligament, together with rotation of the right uterine horn. **(B)** Excised uterus showing marked enlargement and torsion of both uterine horns. **(C)** Gross sectional appearance of the right uterine horn mass showing diffuse dark-red discoloration, consistent with the extensive hemorrhage and necrosis subsequently confirmed histologically. **(D)** Histologic section of the right uterine horn mass, consistent with leiomyoma (hematoxylin and eosin, original magnification ×10).

Histopathologic examination revealed well-circumscribed, unencapsulated spindle cell neoplasms composed of interlacing streams and bundles of cells with oval to elongate nuclei, mild anisocytosis and anisokaryosis, and no mitotic figures in 10 high-power fields, consistent with leiomyoma. The same diagnosis was obtained for both uterine horns and for the vaginal mass (Figure 4D). The right uterine horn mass additionally showed extensive hemorrhage and necrosis, consistent with ischemic injury secondary to torsion.

The dog recovered uneventfully from anesthesia and surgery and was discharged without complication. At the 9-month follow-up examination, the dog remained clinically well, with no evidence of recurrence.

## 3 Discussion

The cumulative tumor burden of the bilateral uterine horn leiomyomas was presumed to have progressively increased uterine weight, resulting in elongation and laxity of the broad ligaments and predisposing the uterus to rotational instability. A similar association between uterine masses and torsion has been reported in dogs with leiomyosarcoma and lipoma (2, 5). The present case suggests that mass-related biomechanical alterations, rather than malignant histologic behavior, may be sufficient to predispose the uterus to torsion.

The marked rotational asymmetry, with approximately 1,080° on the right against 270° on the left, reflects the substantially greater size of the right horn and resembles the intertwined pattern described in the only previous canine report of bilateral uterine horn torsion (7). It is presumed that the heavier right horn underwent primary torsion and that the resulting displacement, together with the marked ligamentous laxity, permitted secondary rotation of the left horn. This sequence cannot be confirmed from static imaging and surgical findings, although the extensive hemorrhage and necrosis identified histopathologically in the right uterine horn were consistent with more severe vascular compromise on that side. In contrast to the previous canine report, no gross rent of the broad ligament was identified (7), suggesting that ligamentous elongation and laxity alone may be sufficient to permit this pattern.

The marked degree of rotation may have been influenced by progressive vascular compromise. In torsion, lymphatic and venous outflow is affected first, resulting in congestion, edema, and enlargement, whereas arterial inflow becomes impaired at a later stage, leading to ischemia and necrosis (12). The resulting increase in uterine volume and weight may have further promoted rotational instability, potentially contributing to the severe right-sided torsion and the subsequent bilateral involvement.

Radiography has limited value in confirming uterine torsion, and the findings may be misinterpreted as pyometra or other uterine disease (13). Although Doppler ultrasonography may demonstrate a whirl sign in some cases (13), this finding is inconsistent, and CT may be the most useful modality for preoperative confirmation.

In the present case, CT provided three complementary findings. First, bilateral whirl signs were identified, the right being more pronounced and corresponding to the greater degree of rotation observed at surgery. The whirl sign represents twisting of the vascular pedicle, broad ligament, and surrounding fat around the torsion axis. Similar signs have been described in dogs with mesenteric volvulus, splenic torsion, and renal torsion (14–16). In a review of 18 non-gravid women with uterine torsion who underwent CT or magnetic resonance imaging, the whirl sign was identified in eight cases, resulting in a reported sensitivity of 44.4% and a positive predictive value of 100% (17). To the authors’ knowledge, this is the first report of bilateral CT whirl signs in canine uterine torsion. Second, CT directly demonstrated spiral rotation of both uterine horns. The right horn showed more extensive rotation and was twisted around the left horn, closely corresponding to the surgical findings of approximately 1,080° rotation on the right and 270° rotation on the left. Third, the enhancement patterns suggested different degrees of vascular compromise and ischemic injury between the two uterine horns. The absent central enhancement of the right uterine mass was consistent with ischemic necrosis, which was subsequently confirmed histopathologically, whereas the minimal central enhancement of the left mass suggested less severe vascular compromise. The thin peripheral rim enhancement of both masses may have represented obstructed peripheral veins or dilated lymphatic vessels associated with edema (11), or residual collateral capsular perfusion (10). The more pronounced enhancement defect of the right uterine mass corresponded to the greater extent of hemorrhage and necrosis identified histopathologically. In addition, the focal hyperattenuating area measuring 54 HU on unenhanced CT was considered to represent intralesional hemorrhage, consistent with the histopathologic findings.

Of the features reported in a human series of subserosal leiomyoma torsion (10), three were present in this case: poor central enhancement, thin rim enhancement, and ascites. The dark fan sign was not identified, most likely because the present case involved intramural leiomyomas and torsion of the entire uterine horn rather than a pedunculated subserosal leiomyoma.

En bloc ovariohysterectomy without prior detorsion has been performed in canine uterine torsion with ischemic necrosis, on the rationale that untwisting devitalized tissue may release thrombi and inflammatory mediators into the systemic circulation (2, 5). The same principle is well established for splenic torsion, in which the release of accumulated toxins may additionally have cardiodepressant effects (18). The CT findings of central non-enhancement and peripheral rim enhancement suggested substantial vascular compromise and supported the preoperative decision to perform en bloc ovariohysterectomy without prior detorsion. The favorable outcome, with the dog remaining clinically well 9 months after surgery, is consistent with the good prognosis reported after prompt excision (3).

From a clinical perspective, CT should be considered in the diagnostic workup of non-gravid dogs with large uterine masses, particularly when marked mass effect or suspected vascular compromise raises concern for uterine torsion. This report has some limitations. The proposed pathomechanism and sequence of rotation are inferential and cannot be verified from a single case. In addition, the diagnostic performance of these CT findings in canine uterine torsion has not been validated and requires evaluation in larger case series.

In conclusion, this is the first report of asymmetric bilateral uterine horn torsion secondary to benign leiomyomas in a non-gravid dog. Bilateral whirl signs, spiral rotation of both uterine horns, and asymmetric enhancement patterns suggestive of vascular compromise and ischemic injury enabled a confident preoperative CT diagnosis, and en bloc ovariohysterectomy without prior detorsion was performed successfully with a favorable long-term outcome. This case demonstrates that even benign uterine neoplasia may reach a cumulative burden sufficient to precipitate bilateral torsion, and it highlights the value of CT in establishing the diagnosis and guiding the surgical approach.

## Data Availability

The original contributions presented in the study are included in the article/supplementary material, further inquiries can be directed to the corresponding author.
